# Comparative Assessment of Familiarity/Novelty Preferences in Rodents

**DOI:** 10.3389/fnbeh.2021.648830

**Published:** 2021-04-13

**Authors:** Annaliese K. Beery, Katharine L. Shambaugh

**Affiliations:** ^1^Department of Integrative Biology, University of California, Berkeley, Berkeley, CA, United States; ^2^Neuroscience Program, Departments of Psychology and Biology, Smith College, Northampton, MA, United States

**Keywords:** social behavior, sociality, partner preference, rat, mouse, degu, prairie vole, meadow vole

## Abstract

Sociality—i.e., life in social groups—has evolved many times in rodents, and there is considerable variation in the nature of these groups. While many species-typical behaviors have been described in field settings, the use of consistent behavioral assays in the laboratory provides key data for comparisons across species. The preference for interaction with familiar or novel individuals is an important dimension of social behavior. Familiarity preference, in particular, may be associated with more closed, less flexible social groups. The dimension from selectivity to gregariousness has been used as a factor in classification of social group types. Laboratory tests of social choice range from brief (10 minutes) to extended (e.g., 3 hours). As familiarity preferences typically need long testing intervals to manifest, we used 3-hour peer partner preference tests to test for the presence of familiarity preferences in same-sex cage-mates and strangers in rats. We then conducted an aggregated analysis of familiarity preferences across multiple rodent species (adult male and female rats, mice, prairie voles, meadow voles, and female degus) tested with the same protocol. We found a high degree of consistency within species across data sets, supporting the existence of strong, species-typical familiarity preferences in prairie voles and meadow voles, and a lack of familiarity preferences in other species tested. Sociability, or total time spent near conspecifics, was unrelated to selectivity in social preference. These findings provide important background for interpreting the neurobiological mechanisms involved in social behavior in these species.

## Introduction

Comparative studies of rodent species that differ in social behavior have yielded important foundations for studying the genetic and neurobiological mechanisms underlying social behavior variation. One such behavior of interest is the tendency to live alone or in groups, and the neurobiological mechanisms underlying sociality in rodents are beginning to be explored (reviewed in Anacker and Beery, 2013; Beery, 2019). In order to understand the behavioral and neurobiological mechanisms that promote group-living across species, it becomes important to carefully consider the nature of relationships between individuals. In particular, mechanisms that support group living may differ when groups are based on selective relationships between group members vs. similar tolerance of both familiar and novel individuals. Despite the relevance of group membership for group structure, familiarity preference has surprisingly not been characterized for many social rodents. We have assessed familiarity preferences in a variety of rodent species that each live in groups but differ in the nature of their social system; here we perform a meta-analysis across one new (this study) and eight published data sets of five rodent species performed following the same protocol.

Laboratory assessments of social behavior are most often conducted in dyads; for example the widely used social interaction test and the sociability test assess the frequency and nature of social interactions between a focal individual and a conspecific (Crawley et al., 2001; File and Seth, 2003). To assess social preferences for familiar or novel individuals, social choice tests are used, with two major design factors: the length of the test is either short (i.e., 5–20 min) or long (>1 h), and tests either provide limited access to conspecifics (e.g., behind a perforated or wire barrier), or direct physical access to conspecifics tethered within a portion of the arena. The most commonly studied rodent species (mice and rats) often exhibit preferences for exploring novel conspecifics more than familiar conspecifics in 10 min sessions, assessed using limited-access novelty preference tests (e.g., Moy et al., 2004; Smith et al., 2015).

In contrast, the partner preference test (Williams et al., 1992b) was developed to assess social preferences for familiar mates that may take longer to manifest. This 3-hour test involves a focal subject that can roam freely between tethered conspecifics. In early descriptions, partner preferences became evident in the 2nd hour and remained stable thereafter (Williams et al., 1992a). While developed and continuously employed to assess selective relationships with mates in prairie voles, variants of this protocol have been used in voles to assess a variety social choices presented: for example a new vs. old partner (Harbert et al., 2020), multiple vs. single stimulus subjects (Ondrasek et al., 2015), and sex of the conspecifics (DeVries et al., 1997; Parker and Lee, 2003). Partner preference has also been assessed in a variety of species from zebra finches to marmosets to gerbils (Kingsbury and Goodson, 2014; Carp et al., 2016; Kowalczyk et al., 2018; Tchabovsky et al., 2019). These behavioral characterizations have laid the foundation for numerous manipulation studies that probe the neurobiological basis of social preferences (Carter et al., 2008; Albers, 2015; Lieberwirth and Wang, 2016; Walum and Young, 2018; Beery, 2019).

The use of the same-sex, peer implementation of the partner preference test (referred to as the same-sex PPT, or as herein: peer PPT) provides a standardized assessment of familiarity preferences which do not emerge during brief preference tests (Beery et al., 2018). We have used the peer PPT in our lab for over a decade in studies of day-length mediated variation in group living (sociality) in meadow voles (Beery et al., 2008b, 2009, 2014; Beery and Zucker, 2010; Anacker et al., 2016a,b; Goodwin et al., 2019; Lee et al., 2019; Lee and Beery, 2021). More recently we have assessed same-sex peer partner preferences in a variety of rodent species that live in groups but differ in the nature of their social system (Table 1). In this manuscript we conduct a comparative analysis of same-sex peer partner preferences in five species: rats, mice, prairie voles, meadow voles, and degus. We aggregated data from unmanipulated subjects in all relevant and comparable studies conducted in our lab, or by lab members together with collaborators working with other species. As part of this analysis we evaluate the consistency of data collected years apart under the same conditions, and we tested meadow voles in two apparatus types to assess the consistency or variability introduced by a change in apparatus structure. Finally, we formally characterize familiarity preferences in the peer PPT in rats—a commonly used laboratory species that does not show long-term indications of familiarity preferences (Schweinfurth et al., 2017), but for which this test had not previously been performed.

**Table 1:** Brief descriptions of the social organization of species described in the peer PPT.

**Species**	**Latin name**	**Group living?**	**Group description**	**Monogamous?**	**Selective preference for familiar peers?**	**Data used in comparative analysis of preferences**
Rat	*Rattus norvegicus*	Yes	Flexible group size depending on local resources. At low density, exclusive male territories overlap multiple females. At high density, rats form mixed sex subgroups within larger groups.	No	No	This study
Mouse	*Mus musculus*	Yes	Flexible group size. When widely dispersed, they exhibit overlapping home ranges and become nomadic after breeding. Feral house mice are often at densities of 10–100 mice/hectare, but surge to 1,000/hectare and have been detected at 100,000/hectare.	No	No	Beery et al., 2018
Prairie vole	*Microtus ochrogaster*	(No)	Females live alone, in male-female pairs, or in small family groups. Females reside in groups 30–70% of the time, typically with undispersed young and occasionally unrelated individuals.	Yes	Yes	Beery et al., 2018; Lee et al., 2019
Meadow vole	*Microtus pennsylvanicus*	Yes	Adult females are territorial during the breeding season. In winter, meadow voles share burrows and cohabit in mixed-sex groups of 3–10 voles.	No	Yes	Anacker et al., 2016b; Lee et al., 2019; this study
Degu	*Octadon degus*	Yes	Individual burrows contain 0–2 males and 1–8 females. Groups exhibit relatively low levels of genetic relatedness, with turnover in group membership both within and across seasons.	No	No (males not tested)	Insel et al., 2020

*Evaluations of group living and monogamy are sourced from Lukas and Clutton-Brock (2013). Narrative descriptions are drawn from the following: Rats: Lore and Flannelly (1977) and Berdoy and Drickamer (2007); Mice: Berdoy and Drickamer (2007). Prairie Vole: Getz et al. (1993). Meadow vole: Madison and McShea (1987); Degu: Ebensperger et al. (2009), Hayes et al. (2009), and Davis et al. (2016). Selective preferences have been examined in males and females of each species except degus, in which tests were performed in females*.

## Materials and Methods

### New Animal Subjects

Long-Evans rats (Charles River, Wilmington, MA) were housed in pairs with a same-sex cage-mate from arrival (d21–28) through peer PPT testing at 75 ± 6 days of age. Rats were housed in 22 × 45 × 25 cm plastic cages on aspen shavings with Envirodri nesting material (Fibercore, Cleveland, OH) and a PVC hiding tube. The light cycle was 12:12 with lights off at 5 p.m. EST. Food (Envigo 2018: Teklad Global 18% Protein Rodent Diet) and water were available *ad libitum*. 12 rats (9 females, 3 males) were used as focal subjects for analysis.

Apparatus comparisons were performed in locally bred meadow voles, housed in pairs with a same-sex cagemate from weaning through peer PPT testing at 80 ± 7 days of age. 8 males were used as focal subjects. Male meadow voles do not show the same day length-dependent variation in social behavior that females do, but exhibit partner preferences in both long and short day lengths (Beery et al., 2009). Meadow voles were housed as described above, but in shorter cages (15 × 45 × 25 cm) with *ad libitum* access to Lab Diet 5015 supplemented with apples and greens. All procedures adhered to federal and institutional guidelines and were approved by the Institutional Animal Care and Use Committee of Smith College.

### Data Sources for Comparative Analyses

Data collected in Long-Evans rats were contextualized in a meta-analysis comprised of same-sex peer PPT data from other species. Social behavior of these species is described in Table 1. Subjects were all control animals from eight data sets from published studies in four additional species for which we have collected data meeting the following inclusion criteria: (1) peer partner preference tests were conducted following long-term (>1 month) cohousing with a same-sex cage-mate, (2) testing was performed in a linear 3-chamber apparatus (earlier tests in our lab used a branched apparatus design), and (3) subjects were unmanipulated controls. Data are included from female degus (Insel et al., 2020), male and female C57BL/6 and C57BL/10 mice (Beery et al., 2018), male and female prairie voles (Beery et al., 2018; Lee et al., 2019; 3 total data sets), and female meadow voles (Anacker et al., 2016b; Lee et al., 2019; 3 total data sets). Female meadow voles were housed in short day lengths, as exposure to winter photoperiods reduces female territoriality and promotes social huddling and group living in this species. All other subjects were housed in long day lengths. We did not include similar data from studies where control subjects were manipulated in any way (e.g., vehicle injected controls, or special diet controls). Similar published data from tests using a branched/non-linear apparatus were not included in analysis, as apparatus configuration influenced time huddling and time in the empty chamber (see Results). Data from all studies that met the inclusion criteria were used to determine species-typical behavior. The complete data set used for comparative assessments is presented in Supplementary Table 1.

### Peer Partner Preference Tests

**Figure 1:**
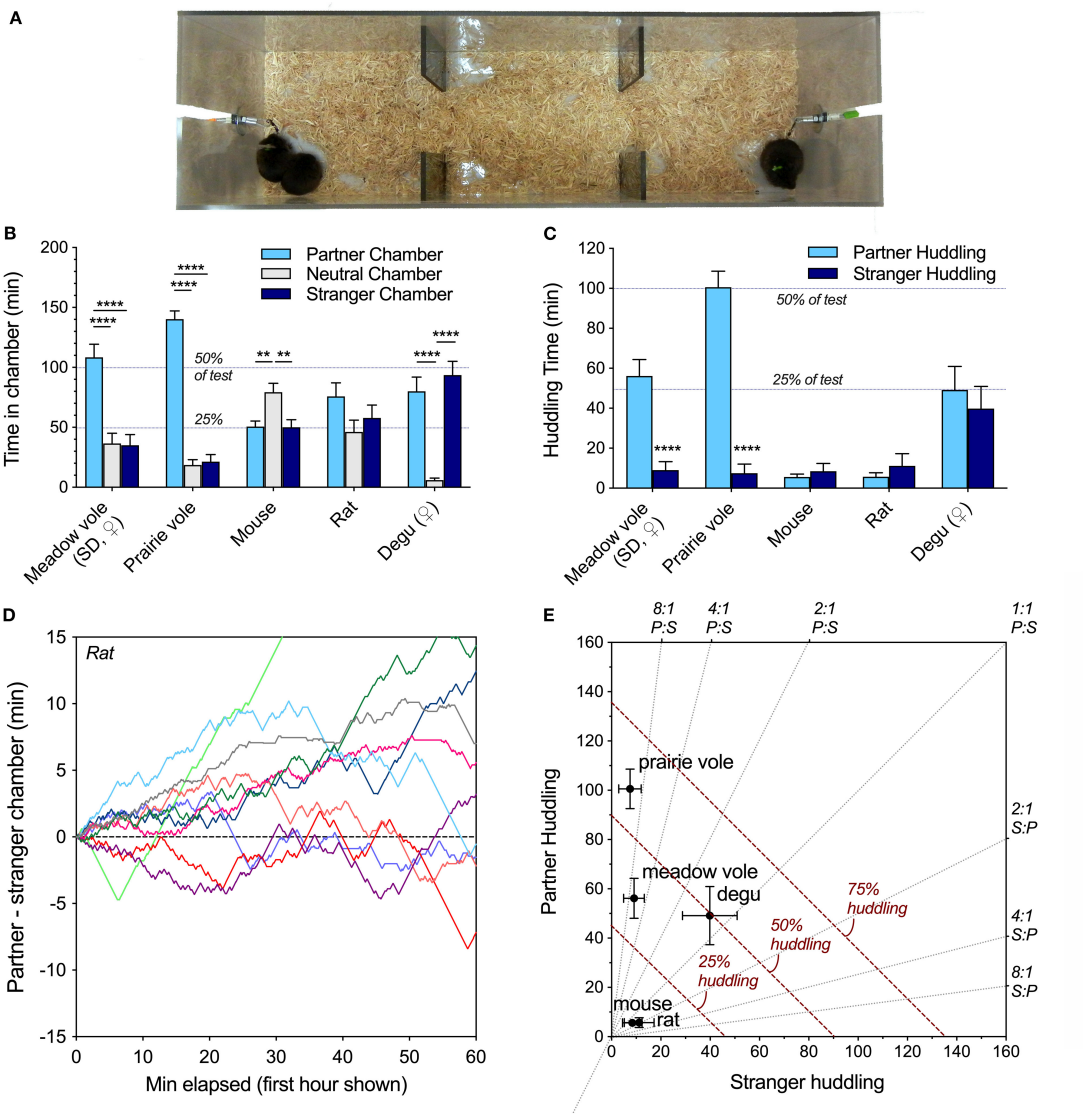
**(A)** Linear partner preference test configuration, shown here with meadow voles. One familiar and one novel individual is tethered at each end. In the peer partner preference test, all animals are of the same sex. **(B)** Species differences were evident in chamber time, with partner selectivity in meadow and prairie voles, preference for the empty chamber in mice, no preferences in rats, and preference for either occupied chamber in degus. **(C)** Selectivity of same-sex peer social interaction across species. Both meadow and prairie voles exhibit selective partner preferences for familiar same-sex peers, while mice, Long-Evans rats, and degus did not. **(D)** Visualization of the time spent in the partner vs. stranger chamber of the PPT apparatus during the 1st h did not reveal the social novelty preferences sometimes displayed by juvenile rats in 10 or 20 min preference tests using barriers. **(E)** An alternate representation of huddling across species highlights variation in both selectivity and total huddling behavior. Gray lines represent level of selectivity. Dashed red lines represent duration of the test spent huddling. Asterisks (^**^ = *p* < 0.01, ^****^ = *p* < 0.0001) represent significant differences within species; see Table 2 for statistical comparisons between species.

Peer PPTs were conducted in a linear 3-chambered apparatus (Figure 1A) in one of two sizes (30 × 30 × 112 cm for rats and degus, 75 × 20 × 30 cm for mice and voles) according to established protocols (e.g., Ahern et al., 2009; Anacker et al., 2016a; Goodwin et al., 2019; Lee et al., 2019). For each test, a familiar same-sex “partner” and a novel same-sex “stranger” conspecific were tethered at opposite ends of the apparatus. A focal rodent was then placed in the neutral center chamber and allowed to roam freely for the duration of the 180 min test. Locations of the partner and stranger were alternated between successive tests, and sessions were video recorded for analysis. Video recordings were scored using custom software (Intervole Timer v1.6; https://github.com/BeeryLab/intervole_timer) without knowledge of the partner and stranger positions.

### Apparatus Comparisons

A new sample of meadow voles was tested in both the linear PPT apparatus (described above), and a branched PPT apparatus consisting of three equal plastic cages (17 × 28 × 12.5 cm): a neutral rear chamber connected by tubes (5 cm diameter, 5 cm length) to two front chambers (Figure 3A). Voles were tested following the protocol above, half with the linear apparatus first, half with the linear apparatus second. Testing with the second apparatus type was conducted 6 days after the first test. The data set used for the apparatus comparison appears in Supplementary Table 3.

**Table 2:** Species-specific measures of peer PPT behavior.

**Species**	**% of test in a social chamber**	**% of test adjacent to a conspecific**	Preference score (100^*^partner adjacent/total adjacent)	**Activity (±SEM)(entries to center chamber)**
Rat	74 ± 5^B^	9 ± 6^C^	61 ± 8^B^	199 ± 21^B^
Mouse	56 ± 4^C^	8 ± 5^C^	55 ± 7^B^	345 ± 21^A^
Prairie vole	90 ± 3^AB^	60 ± 4^A^	93 ± 5^A^	75 ± 13^C^
Meadow vole	80 ± 4^AB^	36 ± 4^B^	87 ± 5^A^	101 ± 16^C^
Degu	97 ± 5^A^	49 ± 7^AB^	52 ± 9^B^	76 ± 23^C^

*Within a column, entries that are not connected by the same letter are statistically different*.

### Data Analysis

Major test outcomes were screened for sex differences in rats, but none were present and the sexes were considered together. Partner preference within each species was defined as significantly more time adjacent to the partner than the stranger in paired *t*-tests. The relative preference for the partner was also expressed as a single preference score, defined as 100^*^(time adjacent to the partner)/(total time adjacent to the partner+stranger) (Beery and Zucker, 2010). Specific test outcomes were compared across species using one-way ANOVA, followed by Tukey's HSD. Statistical analyses were performed in JMP 14 and graphed with GraphPad Prism 9. Results were considered significant at *p* < 0.05, and all tests were conducted two-tailed.

R (The R project for Statistical Computing) was used to extract cumulative chamber preferences from available raw scoring files in rats, mice, and one cohort of prairie voles. Cumulative huddling preferences were extracted for the prairie vole cohort, and partner and stranger huddling were compared at half-hour intervals. Summary measures (partner-stranger (P-S) chamber time, P-S huddling, and preference score) were compared to expected values in the absence of preferences (0 for P-S time; 50% for preference score) using one-sided *t*-tests if data were normally distributed or Wilcoxon signed rank tests if they were not.

## Results

### Rat Preference Behavior

Comparison of time Long-Evans rats spent adjacent to the familiar same-sex partner vs. a novel same-sex stranger in the 3 h peer PPT revealed no significant familiarity or novelty preferences (*t*(11) = 0.82, *p* = 0.43, paired *t*-test). Rats engaged in resting social contact (a.k.a. huddling) with both familiar and unfamiliar peers, but spent an average of <10% of the test huddling (Table 2, Figure 1C). Rats spent 74% of the test in one of the chambers occupied by a conspecific (Table 2, Figure 1B), but there was no significant preference for any chamber over another across the test (*F*_(2, 33)_ = 1.98, *p* = 0.15). Chamber preference (P-S) was also visualized in early test intervals (Figure 1D) and across the test (Supplementary Figure 1). Adult rats did not show a significant preference for either chamber or for resting contact with either stimulus animal at the 10 and 20 min test durations used in social novelty preference tests (P-S chamber time and P-S huddling time tested vs. expected mean values of 0).

### Behavior Within and Across Species

Social preferences were assessed within each species. Meadow voles and prairie voles both demonstrated strong selective preferences for time in contact with a familiar same-sex peer (Figure 1B), as well as time in the partner-occupied chamber (Figure 1C). No other species showed selective same-sex partner preferences. Degus exhibited a preference for either socially occupied chamber over the empty chamber, while mice preferred the empty chamber over either occupied chamber (Figure 1C). When selectivity and total huddling are visualized together (Figure 1D), prairie voles and meadow voles show qualitatively similar but quantitatively different patterns of partner selective behavior, degus show high huddling in the absence of selectivity, and mice and rats showed low levels of huddling and selectivity.

Patterns of behavior were explicitly compared across species using the metrics: percent adjacent to a conspecific, percent in social (occupied) chambers, preference score (percent partner adjacent/total adjacent), and activity. All metrics differed across species (Table 2). Notably, rats and mice spent the least amount of time in social chambers, and much less time in physical contact with either the familiar or unfamiliar conspecific. Degus spent the most time in occupied chambers and huddled as much as prairie and meadow voles, but unlike prairie and meadow voles showed no preference for a familiar over an unfamiliar same-sex peer. Degus, meadow voles, and prairie voles were less active within the apparatus, while mice and rats transitioned between chambers more frequently (Table 2).

### Timeline of Peer Social Preference Development

**Figure 2:**
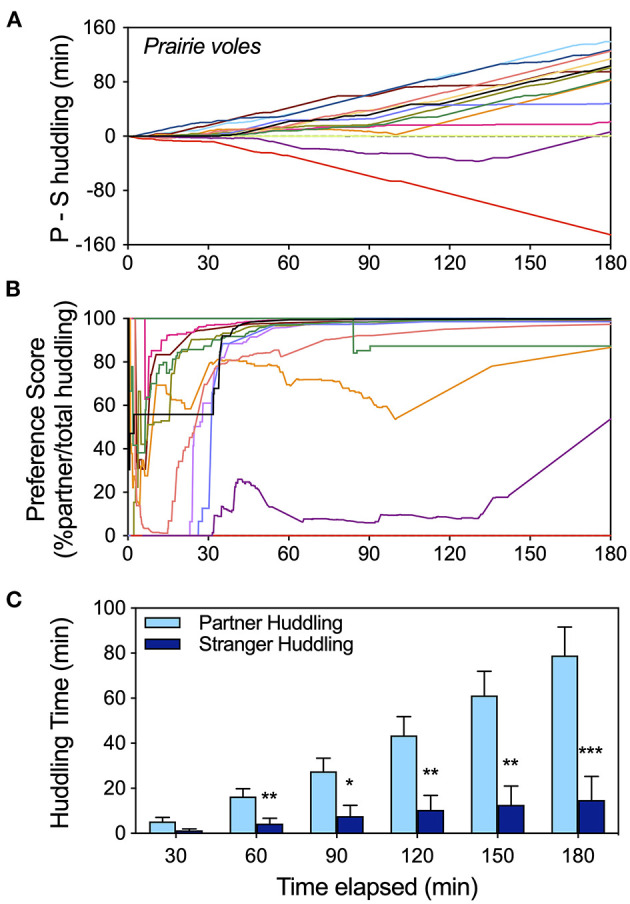
Timeline analysis of the development of huddling preferences in a cohort of female prairie voles using three metrics of preference (data extracted from raw scoring files for all tests labeled “pvole-2” in the Supplementary Data). **(A)** Cumulative display of huddling preferences (partner-stranger huddling) illustrates the trajectory of preference over the course of the test, as well as heterogeneity of behavior. **(B)** The trajectory of preference score stabilizes sooner, and by 1 hour is a good indicator of preference for most subjects. **(C)** Huddling times at half hour intervals illustrate how the full test interval is useful for a larger gap between partner and stranger contact to emerge. Statistical comparisons of partner vs. stranger huddling, as well as differences from expected values for preference metrics (0 for P-S huddling; 50% for preference score) at half hour intervals appear in Supplementary Table 3. ^*^ = *p* < 0.05, ^**^ = *p* < 0.01, ^***^ = *p* < 0.001.

Peer social preference development over time was visualized for a cohort of female prairie voles over the 3 h partner preference test (data set “p-vole2” from Supplementary Table 1). Display of cumulative relative huddling (partner – stranger huddling; Figure 2A) reveals the trajectory of partner huddling predominance over time for the majority of prairie voles, as well as multiple individuals that did not prefer the partner. Preference score (% partner/total huddling; Figure 2B) was extremely variable for the first half hour—during which time relatively less huddling occurs—but tended to stabilize by 1 h, providing an “early” indicator of preference for voles that preferred the partner. Partner preference increased in magnitude and statistical significance over the duration of the test as partner huddling increased faster than stranger huddling, and increased faster during the latter portion of the test (Figure 2C). Preference for the partner vs. the stranger and difference of the composite metrics from mean values representing lack of preference were assessed at half hour intervals to illustrate the different metrics over time (Supplementary Table 3). In contrast to the other two metrics, preference score did not increase in significance after the 1st hour.

### Apparatus Comparison

**Figure 3:**
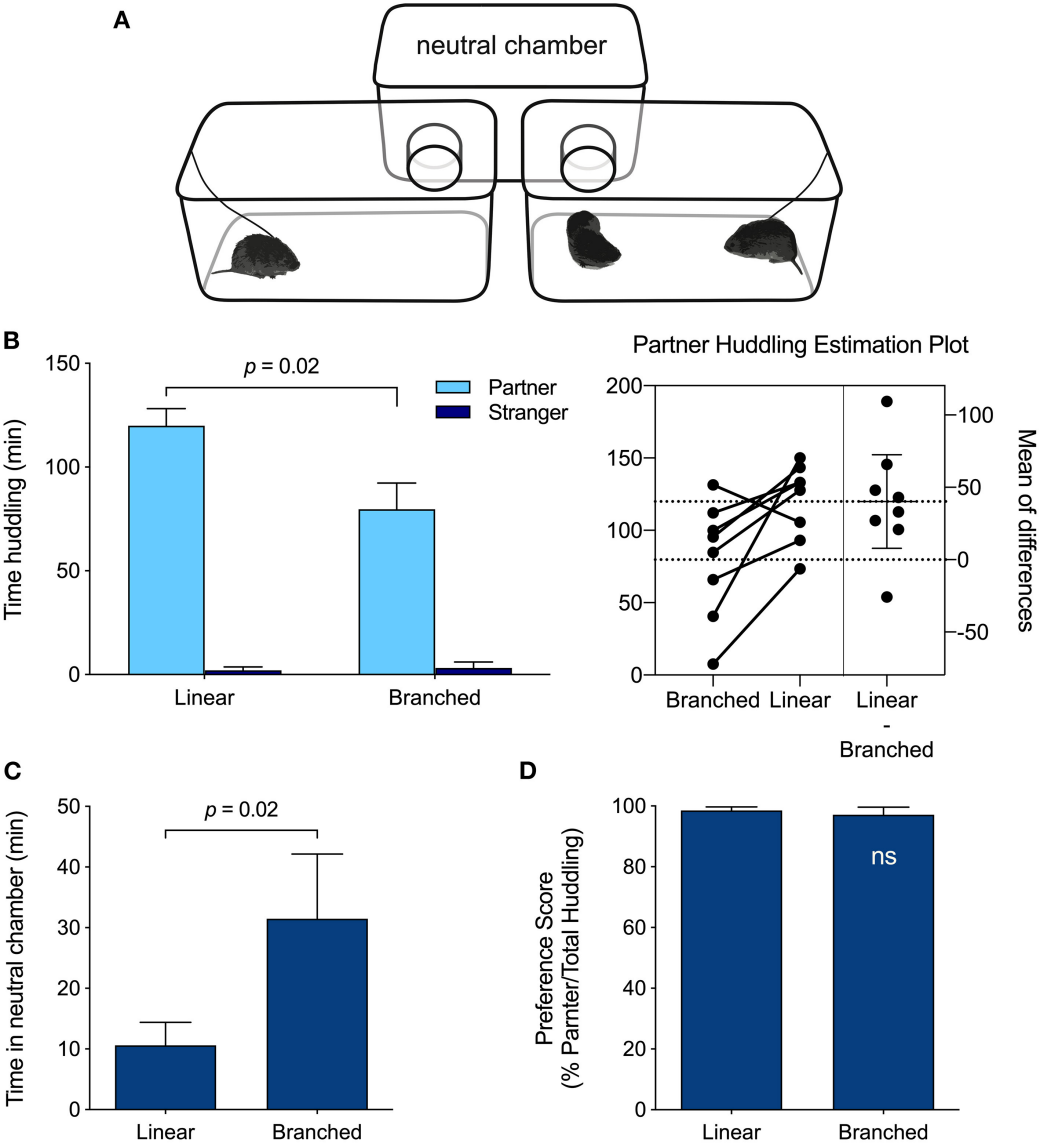
PPT Apparatus effects. **(A)** Branched PPT apparatus configuration. The linear PPT apparatus appears in Figure 1. **(B)** Meadow voles huddled more with their partner when tested in the linear apparatus than in the branched apparatus (*p* = 0.02; paired *t*-test). **(C)** Meadow voles spent less time in the empty, neutral chamber of the linear apparatus than of the branched apparatus (*p* = 0.02; paired *t*-test). **(D)** Despite differences in huddling duration, the relative preference for the partner vs. stranger did not differ across test formats.

Test-retest comparisons of the behavior of meadow voles in the linear (Figure 1A) and branched (Figure 3A) versions of the partner preference testing apparatus revealed differences in total huddling time, but no difference in the relative preference exhibited for partners vs. strangers (partner/total huddling, Figures 3B–D). Meadow voles tested in the linear apparatus spent more time huddling with their partner (*t*(7) = 2.94, *p* = 0.02), and less time in the unoccupied chamber (*t*(7) = 2.95, *p* = 0.02) than voles tested in the branched apparatus (Figure 3C). On the basis of these apparatus differences, comparative analyses across species and samples were restricted to tests that used the linear chamber type.

### Preference Behavior Was Consistent Within Species Tested Even Years Apart

We assessed whether there were differences in same-sex peer PPT behaviors across studies for which we have multiple comparable data sets: prairie voles (3 data sets) and meadow voles (3 data sets). All data are part of Supplementary Table 1. Outcomes assessed were: partner huddling, stranger huddling, activity, and three composite scores: percent in a social chamber (i.e., % P chamber + % S chamber), percent adjacent to either conspecific (i.e., % P huddling + % S huddling), and preference score (100^*^P huddling/P + S huddling). None of these outcomes were significantly different across data sets for either species (all *p* > 0.05; one-way ANOVA). Only two comparisons approached significance: PPT activity in meadow voles, and minutes huddling with the partner in prairie voles (both 0.1 > *p* > 0.05). The lack of significant variation in behavior within species across data sets reinforces the scope of the many highly significant behavioral differences across species described below.

## Discussion

### Rat Social Preference Behavior

The current report places rats within the framework of the peer partner preference test, and illustrates similarities and differences between rats and other rodents tested using this behavioral assay. The finding that rats do not show selective partner preferences for familiar same-sex peers is consistent with a recent report showing that female rats showed no stable relationships with particular group members, either over time or in different social contexts (Schweinfurth et al., 2017). We now conclude that even over the extended intervals that reveal familiarity preferences in meadow voles and prairie voles, rats do not prefer familiar social partners to novel ones. This may be relevant to their flexible group structure, gregariousness, and potential to live in large groups (Berdoy and Drickamer, 2007).

Rats often show a preference for social novelty working harder to access a non-cagemate than a cagemate peer (Hackenberg et al., 2021), and interacting more with unfamiliar conspecifics during short (10–20 min) tests of social behavior (Smith et al., 2015, 2018). Rats did not exhibit novelty preferences at these early timepoints in the present study. This may be due to strain differences (Long-Evans vs. Wistar), age (adult vs. juvenile), or testing conditions (subjects tethered instead of behind a barrier).

### Species Differences

The peer PPT reveals profound differences in familiarity preferences across species, consistent with species-typical variation in social group composition. Mice, like rats, can be found in high density groups in areas of human habitation, and these groups are both flexible and gregarious. Mice form dominance hierarchies based on repeated interactions (reviewed in Beery et al., 2020), but did not display selective preferences for familiar same-sex peers, and exhibited low social contact overall.

Both vole species tested exhibited strong, selective preferences for familiar same-sex peers, despite differences in mating system. Prairie voles typically reside with a mate in socially monogamous partnerships year round, but may also form extended family groups with undispersed offspring, or even multiple breeding females (Getz et al., 1981; Hayes and Solomon, 2004; Madrid et al., 2020). These relationship types are consistent with a high degree of selectivity for familiar individuals, including same-sex peers.

Meadow voles mate promiscuously, but live in groups outside of the breeding season. Groups typically begin with a mother and undispersed offspring, but immigration in the wake of predation leads to a lack of kin structure by mid-winter. Nonetheless, groups remain largely stable, and by late winter/early spring are no longer open to new members (Madison et al., 1984; Madison and McShea, 1987). This relatively closed social structure may support the selective social preferences seen in the peer PPT. Field variation in social behavior is recapitulated under conditions of changing day length in the laboratory, with voles housed in short, winter-like day lengths (10 h light:14 h dark) exhibiting high huddling with familiar partners. Interestingly, while short day meadow voles are highly affiliative toward familiar peers, they are also more tolerant of strangers than are their long day-housed counterparts; female meadow voles housed in long day lengths huddle significantly less than short day females, but when they do huddle, it is even more exclusive to the familiar peer (Beery et al., 2008b). Stranger directed aggression also increases in summer months in the field, and in long day lengths in the laboratory (McShea, 1990; Lee et al., 2019). Thus, both affiliation toward a partner and selective aggression toward a stranger may influence group structure in meadow voles.

In the peer PPT, degus exhibited a high degree of social contact in the absence of familiarity preferences. Social groups in degus are centered around groups of 1–8 females with 0–2 males (Fulk, 1976; Ebensperger and Wallem, 2002; Hayes et al., 2009; Ebensperger et al., 2012), and females exhibit alloparental care (Ebensperger et al., 2002). Group membership is not substantially kin-based (Quirici et al., 2011; Davis et al., 2016), and groups are unstable both within and across seasons (Ebensperger et al., 2009). The high degree of affiliative behavior toward both familiar and unfamiliar conspecifics in the PPT may be a characteristic that aids in supporting dynamic group structure in the wild.

Characterization of peer social preferences provides an important framework for interpreting the results of studies of the neurobiological and physiological underpinnings of social behaviors, and may inform the choice of model organism for studies of selective social relationships. For instance, while mice and rats are by far the dominant mammalian laboratory subjects (Manger et al., 2008; Beery and Zucker, 2011, Supplementary Material), neither species is suitable for studying affiliative relationships. Characterization of the social preferences of additional rodents will enhance this measure of social organization across species. For example, studies are underway of same-sex peer social preferences in naked mole-rats (*Heterocephalus glaber*; M.M. Holmes, personal communication), and social tuco-tucos (*Ctenomys sociabilis*, E.A. Lacey, *personal communication*). This test may also provide a useful field-employable indicator of a fundamental aspect of social behavior. Assessed consistently within a phylogenetic context, preference behavior should also be a useful attribute to relate to other social behavioral and physiological variables.

### Preference Timeline

Partner preference typically becomes evident by 2–3 hours into the partner preference test, and in some data sets may be significant at earlier timepoints. Detailed examination of the timeline of preference in a cohort of female prairie voles tested after long-term cohabitation with a same-sex partner confirmed this understanding in peer PPTs, and illustrates the pattern of social preference. In the first 30–60 min, preference score (relative huddling with the partner/total huddling) was variable, stabilizing by ~60 min as a good early metric of preference and not increasing in significance over the course of the test. In contrast, huddling differences (partner-stranger) and relative huddling (partner vs. stranger huddling) both increased throughout the test. The pattern of preference was consistent, i.e., prairie voles did not switch from preferring novelty to preferring familiarity partway into the test. Rather, most voles showed stable but increasing partner huddling preferences, and huddled more later in the test. This is in line with prior work demonstrating that in 10 min social preference tests, prairie voles did not exhibit novelty preferences (Beery et al., 2018) as other rodents such as mice and rats often do (Moy et al., 2004; Smith et al., 2017).

### Behavioral Consistency

There were no significant differences in peer PPT behavior *within* species across data sets using the same apparatus type, even those collected years apart. This is remarkable for behavioral traits that may vary with ambient conditions (Crabbe et al., 1999). Many studies, including our earlier work, use a branched apparatus for PPTs; direct comparisons of behavior across the apparatuses revealed significant effects of apparatus type, with reduced time huddling and increased time in the empty central chamber. In the linear apparatus, the focal vole is less separated from conspecific voles in the empty chamber, which may contribute to increased huddling. These differences led us to include only studies using the linear apparatus in the comparative data set. However, despite apparatus effects on huddling times, the relative extent of partner preference (i.e., partner/total huddling) did not differ across apparatus types. Instead, the existence and extent of this preference appears to be a robust, characteristic behavior suitable for comparison across species or populations, even when testing details are varied.

### Comparative Behavioral Assessments Elucidate Underlying Mechanisms

Comparative assessments of behavior across species enable better understanding of sources of variation in traits. Uniform comparisons of behavior can be challenging, because when behaviors (e.g., displays, vocalizations, etc.) vary substantially between species, it becomes difficult to identify specific features that can be compared. For those behaviors that are general and quantifiable, it becomes possible to identify meaningful points of convergence and divergence. Collected in a consistent manner across a phylogenetic tree, behavioral traits can be overlaid on neurobiological, physiological, and genetic data to improve our mechanistic understanding of behavior. This has been undertaken principally with physiological rather than behavioral measures, but when behavioral traits lend themselves to comparison (as in the case of familiarity preference behaviors) they can be fruitful targets of investigation.

For example, comparisons of neuropeptide receptor binding patterns have been made across mole-rat species differing in social organization (Kalamatianos et al., 2010; Coen et al., 2015). The genetic bases of both burrow architecture and parental behaviors have been assessed across species and hybrids of *Peromyscus* mice (Weber et al., 2013; Bendesky et al., 2017). The neural basis of convergence in parental behaviors has been examined across behaviorally diverse poison frogs (Fischer et al., 2019), as has the genetic and electrophysiological basis of convergent electrical signaling across electric fishes (Gallant et al., 2014; Swapna et al., 2018). More commonly, comparisons of smaller numbers of species provide a starting place for understanding the mechanisms underlying behavioral diversity, e.g., of mating systems in voles (Young, 1999), docility and anxiety in *Peromyscus* mice (Martin et al., 2007), group living in South American tuco-tucos (Beery et al., 2008a), foot flagging in tropical frogs (Mangiamele et al., 2016), and countless other behaviors.

Studying diverse species is important to determine the variety of pathways supporting behaviors, as well as the generalizability or translatability of findings across species. The integrative study of animal behavior has increasingly begun to merge proximate/mechanistic and ultimate/evolutionary approaches to the study of behaviors, particularly through comparative work and research in non-traditional model organisms (Donaldson, 2010; Phelps et al., 2010; Hofmann et al., 2014; Rubenstein and Hofmann, 2015; Taborsky et al., 2015). Familiarity preference is a social behavioral trait that differs markedly among species tested to date, and provides an easily tested and useful attribute to include in comparative assessments.

## Data Availability Statement

The original contributions presented in the study are included in the article/Supplementary Material, further inquiries can be directed to the corresponding author.

## Ethics Statement

All procedures adhered to federal and institutional guidelines and were approved by the Institutional Animal Care and Use Committee of Smith College.

## Author Contributions

KS conducted and scored partner preference tests in rats. AB oversaw research, analyzed new and comparative data sets, and wrote the manuscript. Both authors reviewed the manuscript.

## Conflict of Interest

The authors declare that the research was conducted in the absence of any commercial or financial relationships that could be construed as a potential conflict of interest.
