# Supplementary material for: Comparative Assessment of Familiarity/Novelty Preferences in Rodents
Source: Front Behav Neurosci. 2021 Apr 13;15:648830. doi: 10.3389/fnbeh.2021.648830 (PMC8076734; doi:10.3389/fnbeh.2021.648830)
Supplement: Supplementary file 2 [file Image_1.PDF]

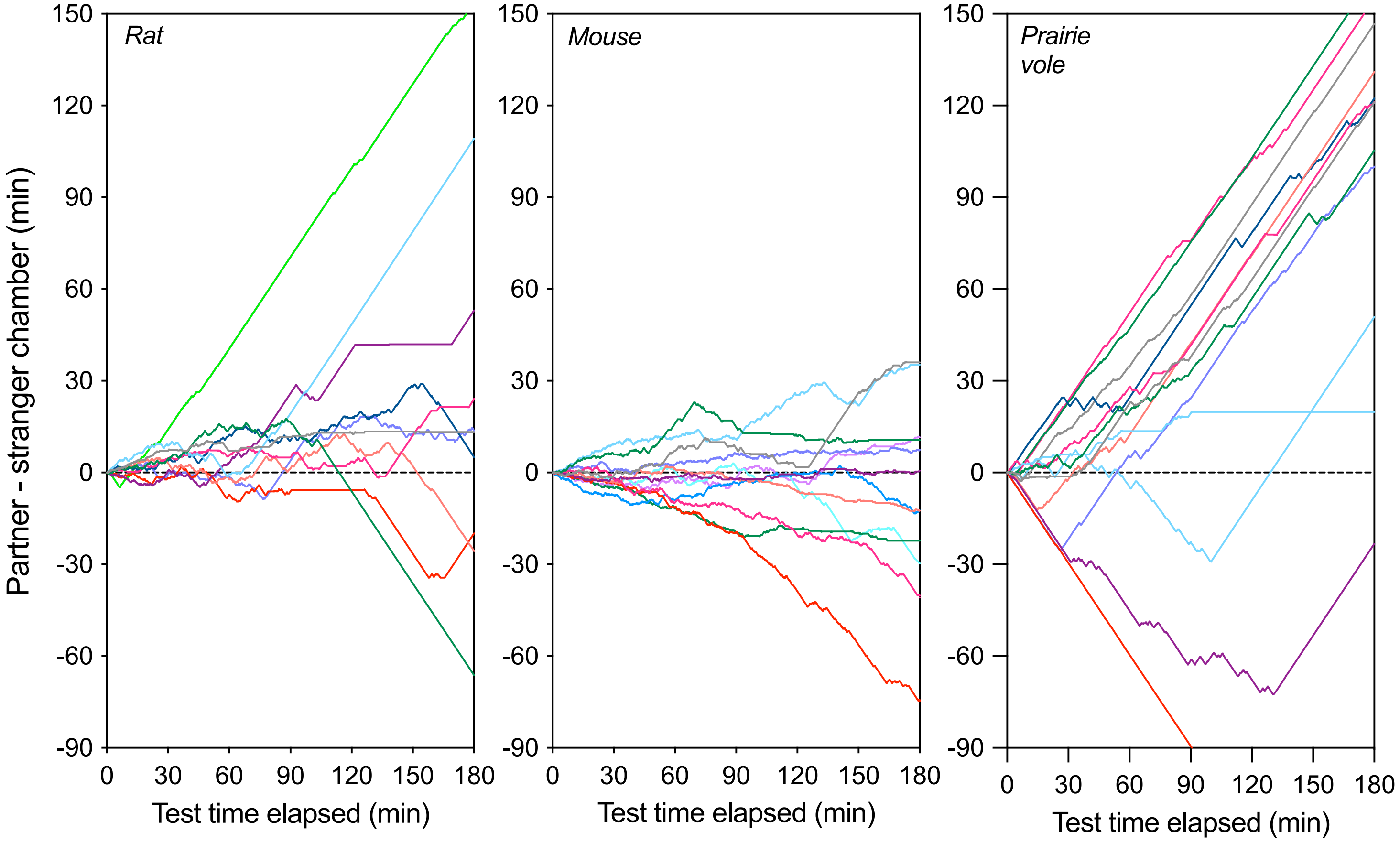


**Figure S1.** Chamber preferences visualized across the full PPT interval for three species (cohorts and individuals represented are marked in the supplementary data file).
